# Ecological Factors Driving Avian Influenza Virus Dynamics in Spanish Wetland Ecosystems

**DOI:** 10.1371/journal.pone.0046418

**Published:** 2012-11-12

**Authors:** Elisa Pérez-Ramírez, Pelayo Acevedo, Alberto Allepuz, Xeider Gerrikagoitia, Anna Alba, Núria Busquets, Sandra Díaz-Sánchez, Vega Álvarez, Francesc Xavier Abad, Marta Barral, Natàlia Majó, Ursula Höfle

**Affiliations:** 1 Instituto de Investigación en Recursos Cinegéticos (IREC), UCLM-JCCM-CSIC, Ciudad Real, Spain; 2 Biogeography, Diversity and Conservation Research Team, Department of Animal Biology, Faculty of Sciences, University of Malaga, Málaga, Spain; 3 Centre de Recerca en Sanitat Animal (CReSA), UAB-IRTA, Bellaterra, Barcelona, Spain; 4 Departament de Sanitat i Anatomia Animals, Facultat Veterinària, Universitat Autònoma de Barcelona, Bellaterra, Barcelona, Spain; 5 NEIKER-Instituto Vasco de Investigación y Desarrollo Agrario, Animal Health Department, Derio, Bizkaia, Spain; University of Georgia, United States of America

## Abstract

Studies exploring the ecological interactions between avian influenza viruses (AIV), natural hosts and the environment are scarce. Most work has focused on viral survival and transmission under laboratory conditions and through mathematical modelling. However, more integrated studies performed under field conditions are required to validate these results. In this study, we combined information on bird community, environmental factors and viral epidemiology to assess the contribution of biotic and abiotic factors in the occurrence of low pathogenic AIV in Spanish wetlands. For that purpose, seven locations in five different wetlands were studied during two years (2007–2009), including seven sampling visits by location. In each survey, fresh faeces (n = 4578) of wild birds and water samples were collected for viral detection. Also, the vegetation structure, water physical properties of wetlands, climatic conditions and wild bird community composition were determined. An overall AIV prevalence of 1.7%±0.4 was detected in faecal samples with important fluctuations among seasons and locations. Twenty-six AIV were isolated from the 78 RRT-PCR positive samples and eight different haemagglutinines and five neuraminidases were identified, being the combination H3N8 the most frequent. Variation partitioning procedures identified the combination of space and time variables as the most important pure factor – independently to other factors – explaining the variation in AIV prevalence (36.8%), followed by meteorological factor (21.5%) and wild bird community composition/vegetation structure (21.1%). These results contribute to the understanding of AIV ecological drivers in Spanish ecosystems and provide useful guidelines for AIV risk assessment identifying potential hotspots of AIV activity.

## Introduction

Avian influenza viruses (AIV) belonging to Influenzavirus A genus infect a broad variety of vertebrates, mainly avian species, but also diverse mammals including humans [1]. Most strains of AIV are low pathogenic (LPAIV) and cause minimal disease in domestic and wild birds, but infection results in high levels of virus excretion, efficient transmission among susceptible hosts and perpetuation of the virus. In the last twenty years, LPAIV of the H5 and H7 subtypes have become an important concern for public and animal health due to their potential to mutate to highly pathogenic AIV (HPAIV) and to seriously affect both human and animal health [2]. Many wild bird species may harbour LPAIV, but Anseriformes (ducks, geese and swans) and Charadriiformes (gulls, terns and shorebirds) are the main hosts and reservoirs of AIV. However, the presence of AIV has occasionally been reported in other species that use wetlands, including birds in the orders Ciconiiformes, Gaviiformes, Gruiformes, Pelecaniformes, Podicipediformes and Procellariformes [3].

Currently, there is limited scientific information about the interface between the ecology and the epidemiology of AIV in wild birds and their persistence in natural ecosystems [4]–[6]. Recent studies have examined the capacity of survival of AIV in experimental infections [7], in water [8], [9] and on diverse inanimate surfaces under different in vitro conditions [10]. In addition, the main routes and enhancing conditions of AIV transmission have been studied experimentally and using mathematical models [11], [12]. However, more integrated virological studies are required to validate mathematical models as well as to identify other potential variables and interactions that may influence AIV infectivity in more complex field conditions. Numerous authors have underlined the relevance of combining information about bird community ecology, environmental factors and viral epidemiology in relation to risk assessment and development of control strategies for AIV and other birdborne diseases [4], [13], [14]. This integrated approach has been applied to the study of AIV persistence over time in African wetlands [15], [16]. In these studies, a relation between host community dynamics and virus ecology is presented with potential for a predictive approach at least at local scale [15]. Gaidet et al. [16] have very recently used a continental scale dataset to test the relative role of several ecological factors on AIV prevalence under the specific climate and seasonality conditions of tropical ecosystems. However, due to numerous differences between tropical and temperate regions, mechanisms underlying main ecological drivers on AIV patterns are expected to differ considerably, especially in relation to environmental transmission (e.g. through long term persistence of the virus in the environment). As far as we know, to date, no field study has been designed to explore the effects of a wide variety of biotic and abiotic factors such as climatic conditions, water characteristics and food and shelter availability for birds on AIV prevalence in wetlands of temperate regions.

The Iberian Peninsula is strategically located in the Mediterranean area in relation to migratory flyways and many of its wetlands are important reserves and major stop-over points for breeding and migratory birds between Eurasia and Africa. For this reason, Spanish wetlands are important locations for disease surveillance and of great interest for the study of LPAIV epidemiology under Mediterranean conditions [17], [18]. In fact, the presence of AIV subtypes has been documented in different wild bird species in wetlands from the Basque Country (North of Spain; [19]), Catalonia (North-East of Spain; [17]) and Castilla-La Mancha (South-Central Spain; [18]). These recent descriptive AIV studies have been used as starting point of the present study with the aim of moving forward in the research of the ecological drivers of LPAIV epidemiology in Spanish wetlands. Identification of ecological factors underlying this complex host-pathogen system may enhance understanding of pathogen dynamics and resource allocation planning [12], [20].

The aim of this study was to determine the influence of a wide range of ecological factors (climatic conditions, density and diversity of wild birds, water physico-chemical properties and shelter and food availability) on AIV dynamics in Spanish wetlands under field conditions. The analysis of the field data in this study represents an approach that adds novel information to the last generated mathematical models [4], [12].

## Materials and Methods

### A. Study area

**Figure 1:**
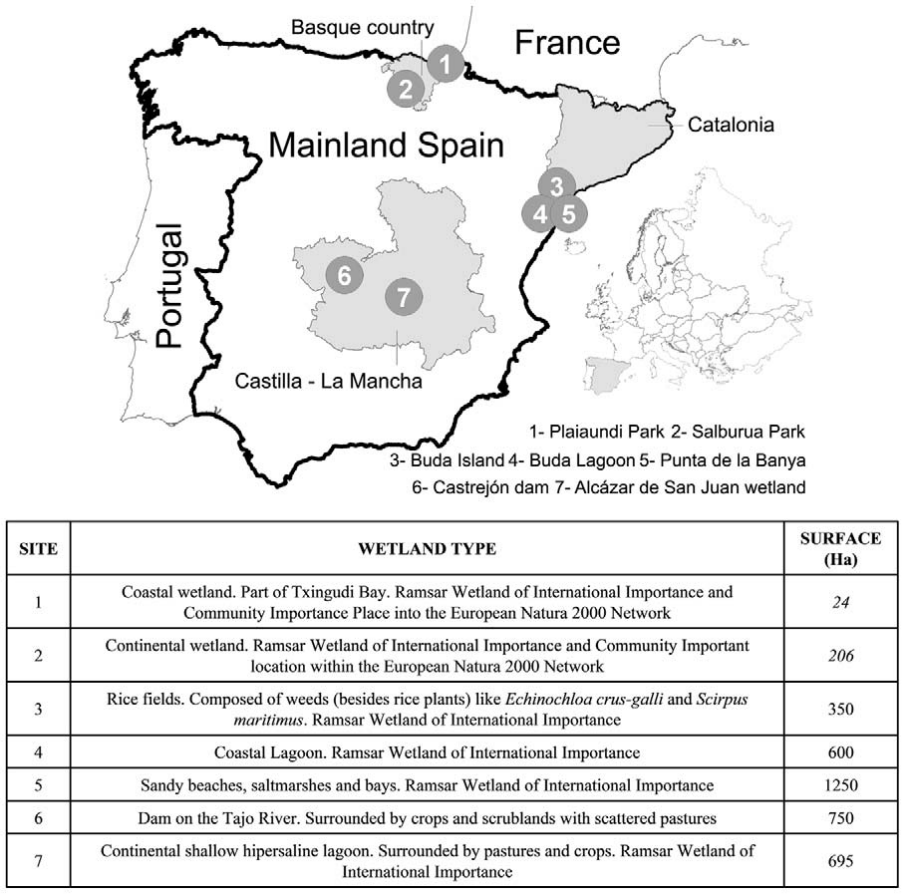
AIV ecology in Spanish wetlands: study area and sampling locations.

The study was carried out in seven sampling locations from five Spanish wetlands (see Figure 1). These wetlands are protected areas that are considered important reserves for migratory and breeding birds in the Western Mediterranean and are representative of the main type of wetland ecosystems that exist in Spain. The sampling localities in northeastern Spain (Catalonia) are part of the Ebro Delta natural park but represent three well differentiated habitats, including rice fields, salt marshes and fresh water lagoons. Type, size and location of the study sites are described in Figure 1.

### B. Sampling period

Faecal sampling and collection of environmental and bird population data were carried out from winter 2007 to autumn 2009. According to host ecology of the most important AIV reservoirs (Anseriformes), three sampling periods corresponding to autumn migration and wintering (AM/W; from August to January), spring migration (SM; from February to April) and breeding/moult (BM; from May to July) were established. In total, 7 sampling visits were carried out to each wetland (AM/W 2007, SM 2008, BM 2008, AM/W 2008, SM 2009, BM 2009 and AM/W 2009).

### C. Sample collection and virological analysis

#### C.1. Faecal sampling

In every wetland, suitable sampling sites (where wild birds aggregate to feed or rest) were identified prior to sample collection with the help of ornithologists. Only faeces that appeared freshly passed as judged by appearance of surface, colour and moisture were sampled. We obtained fresh droppings in every visit, excluding two visits to the Castrejón dam wetland where no fresh samples could be obtained.

**Table 1:** Explanatory variables used to model AIV prevalence in Spanish wetlands.

Factor	Code of predictor	Definition
Water characteristics	Mean temperature	°C
	Mean pH	
	Mean conductivity	Ms/cm
	Mean turbidity	FTU
Wild bird communities	Census of wild birds*	Total number of wild birds
	Species richness	Number of wild bird species
	White storks (*Ciconia ciconia*)^1^	Density of White storks (ind/km^2^)
	Flamingos (*Phoenicopterus ruber*)^1^	Density of Flamingos (ind/km^2^)
	Others*	Density of wild birds other than Anseriformes, White storks and Flamingos
	Anseriformes	Density of Anseriformes (ind/km^2^)
	Dabbling ducks	Percentage of Anseriformes that are dabbling ducks
	Dabbling ducks + Flamingos^2^	Percentage of Anseriformes and Flamingos
Meteorological data	Mean monthly humidity at 00 h	% RH
	Mean monthly humidity	Average of mean highest and lowest daily humidity (% RH)
	Mean monthly temperature	°C
	Monthly mean highest daily temperature MMHDT	°C
	Monthly mean lowest daily temperature MMLDT	°C
	Average of MMHDT and MMLDT	°C
	Total monthly rainfall	mm ×10
Vegetation structure	Vegetation thickness (%)	Percentage of the transect length offering shelter to birds
	Vegetation thickness in the lake shoreline (%)	Percentage of transects with shelter on 2m from the lake shoreline
	Feeding grounds (%)	Percentage of feeding grounds in 1km radius around the wetland

Variables marked with * were excluded as highly correlated (Spearman's coefficient ≥ |0.6|) with other variables within their factor.

1 Flamingos and White storks were considered separately due to previous epidemiological data obtained in the area that identified these species as important AIV carriers [17], [18].

2 In this index we grouped dabbling ducks and flamingos due to similar feeding habits, as both avian groups feed on surface water, which has been identified as a risk factor in AIV epidemiology [24].

In total, 4578 samples were collected. Approximately 0.1 g of faecal matter were placed in 1 ml of transport medium (Hanks or PBS buffered saline solution with 10% glycerol plus antibiotics and antifungal agents [1000 U/ml penicillin, 1000 U/ml streptomycin, 100 μg/ml gentamicin and 50 μg/ml nystatin]) and transferred in a refrigerated container (4 to 10°C) to the corresponding diagnostic laboratories in less than 24 h. Upon arrival, samples were stored at −80°C until analysis. In the case of Basque Country wetlands, complete faeces were collected and maintained in refrigeration without transport medium due to proximity of the sampled wetlands to the laboratory (less than 2 h).

#### C.2. Water sampling and processing

Two litres of wetland water were collected in every visit at two different points of the water body (4 litres for every location and visit). Temperature, pH, conductivity and turbidity of water were registered directly in the field (Table S1) by means of portable equipment (Hanna Instruments S.L., Eibar, Spain). Water samples were kept between 4 and 10°C during transport to the laboratory and stored at −80°C upon arrival. For AIV detection, each water sample was processed for concentration and precipitation of any potential viral material using PEG6000 as previously described [21]. Briefly, 2 litres of water were mixed with 200 g of PEG (Polyethylene glycol, 6000 BioUltra, Sigma-Adrich, Madrid, Spain) and adjusted to 0.3 M NaCl concentration. After complete mixing, the whole volume was kept under gentle stirring at 4–8°C overnight. Stirred samples were then centrifuged at 4°C at 3000 g for 70 min. The resulting pellet was resuspended in Eagles Essential Medium (EMEM, Sigma-Aldrich, Madrid, Spain) volume of 10 to 20 ml, depending on the size of the pellet, and frozen at −80°C until further analysis for virus detection.

#### C.3. Virus detection

Molecular analyses were performed in three different laboratories located in the three sampled regions, assuring minimal transport time of samples from the field. In laboratories located in Catalonia and Basque Country samples were screened following a TaqMan real time RT-PCR (RRT-PCR) specific for the matrix gen (gene M) in the segment 7 of AIV using primers previously described [22]. Viral RNA was extracted using QIAamp Viral RNA extraction kit (Qiagen, Hilden, Germany) following the manufacturer's instructions. Amplification was performed using a one-step RT-PCR kit (Life Technologies-Applied Biosystems, California, USA) following the manufacturer's instructions in Fast7500 equipment (Life Technologies-Applied Biosystems, California, USA) for 40 cycles.

In the corresponding laboratory in Castilla-La Mancha, RNA was extracted using commercial kits (High Pure RNA isolation kit, Roche Diagnostics, Germany) according to the manufacturer's instructions. AIV was detected using a RRT-PCR assay targeting the matrix gene as described by Ward et al. [23] with modifications in the probe sequence as recommended by Munster et al. [24]. Amplification and detection was performed on an iQ5 real time detection system (BioRad) with a TaqMan EZ RT-PCR Core Reagents kit (Life Technologies-Applied Biosystems, California, USA).

Although two different protocols were used for RNA extraction and RRT-PCR, equal sensitivity and specificity in AIV detection was assured by means of an interlaboratorial assay controlled by the Spanish National Reference Laboratory.

In all cases, pools of five individual samples were processed and upon identification of any AIV positive pool, RNA extraction and RRT-PCR procedures were repeated for the individual samples within each positive pool. Individual RRT-PCR positive samples were subsequently used for virus isolation.

#### C.4. Virus isolation and characterization

For AIV isolation from RRT-PCR positive samples, 100–200 μl of the original material were inoculated into the allantoic cavity of 9–11 day-old embryonated specific pathogen free chicken eggs following OIE recommendations [25]. The allantoic fluid was harvested as the embryo died or after 7 days if the embryo was still alive. RNA from allantoic fluid was extracted using a commercial kit (QIAampViral RNA1Mini Kit, Qiagen, Hilden, Germany) and RRT-PCR to detect AIV matrix gene was carried out [22]. When no AIV was detected, the allantoic fluid was passaged twice in embryonated chicken eggs.

#### C.5. Subtype identification

The haemagglutinin (HA) and neuraminidase (NA) were identified, when possible, by sequencing or by direct PCR techniques following the protocols described by Hoffmann et al. [26], Alvarez et al. [27], Gall et al. [28], Tsukamoto et al. [29] and Fereidouni et al. [30] with minor modifications. Comparisons with published sequences were performed by sequence homology searches at the network server of the National Centre for Biotechnology Information (NCBI) using BLAST (http://www.ncbi.nlm.nih.gov/BLAST/). The pathogenicity of the H5 and H7 isolates was determined by the study of the sequence at the HA cleavage site.

### D. Ecological data

#### D.1. Census data for aquatic birds

Although AIV transmission and perpetuation in an ecosystem is dependent on many different factors, abundance and density of susceptible hosts is a key factor in the epidemiology of the virus [3].

To estimate wild bird species richness and abundance, focal counts of wild birds were undertaken during morning hours in each visit. A point counting approach with several experienced observers was used, in accordance with the waterbird monitoring protocols proposed by the Agreement on the Conservation of African-Eurasian Migratory Waterbirds [31]. In the case of the large Castrejón dam, a combined wade-rush/point counting method (using a small motorboat) with numerous experienced observers at different points around the wetland was used (for details about counting methods see [32], [33]). Briefly, countings lasted 30 min and included all aquatic birds within a 200 m radius of each observer.

These data were used to estimate density of Anseriformes, White storks, Flamingos and other wild birds as well as to quantify the percentage of dabbling ducks and the number of wild bird species (see Table 1).

#### D.2. Vegetation structure on the lake shoreline: shelter availability

The availability and spatial disposition of food and shelter determines the abundance and aggregation of wild birds in and around wetlands [34]. Areas in which shelter is abundant will lead to higher concentrations of birds, and thus, higher risk of pathogen transmission [35]. As a measure of shelter availability, the vegetation cover on the shoreline was characterized in every visit and location. Four to five transects of 100 m, perpendicular from the lake shoreline and evenly distributed along the whole water body perimeter were conducted. Shelter availability was assessed using the line intercept method [36] in order to estimate the percentage of the transect length offering vegetation shelter to birds (i.e. areas densely covered by plants mainly of genus *Tamarix*, *Juncus*, *Erica*, *Typha*, *Paspalum*, *Echinochloa*, *Phragmites* and *Scirpus*, depending on the sampling site) and the percentage of transects with shelter on the waterbody shoreline (at no more than 2 m distance from the shoreline).

#### D.3. Land use: food availability

Aggregation of birds in foraging areas greatly increases the contact rates among individuals, creating ideal conditions for disease transmission [35]. To evaluate the potential role of this factor, we characterized agricultural use of patches around wetlands. Using geographic information system, we created land use maps encompassing patches 1 km around the wetland perimeter. These patches were visually inspected in every visit recording both the agricultural use and its stage of development. We considered as potential food source for birds those uses providing either grain (i.e. ripe or recently harvested wheat, barley, rice or corn fields) or fresh green (i.e. alfalfa, recently germinated barley or wheat, growing green grass). These data were used to calculate the percentage of the land around the wetland offering food for the birds (see Table 1).

#### D.4. Meteorological data

Weather conditions have been recognized as an important parameter with regard to migratory bird movements and AIV environmental survival [8], [18], [37]. To evaluate the possible influence of meteorology on AIV dynamics in our study area, information on daily mean temperature, relative humidity and rainfall for each sampling month was obtained from the National and Regional Meteorological Services for the nearest stations to the wetlands included in the study. With this raw information several indexes were calculated as potential determinants of AIV persistence (see Table 1): mean monthly temperature, monthly mean of the lowest daily temperature, monthly mean of the highest daily temperature, the average of mean monthly highest and lowest temperature, mean monthly humidity at 00:00 h, average of mean monthly highest and lowest humidity and total monthly rainfall.

### E. Statistical analysis

The 95% confidence intervals for the proportion of positive samples detected in each location and period was estimated by the exact binomial method with Epicalc 2000 (Brixton Health).

The effect of the ecological factors on AIV positivity in every wetland and visit was assessed using logistic regression [38]. The dependent variable was the number of samples positive for AIV detection in each wetland and sampling period (in relation to the sample sizes). To manage the potential pseudo-replication, “locality”, “season” and “year” were considered as fixed variables (maintained in all stepwise regressions). Indexes described in Table 1 were included as covariables. We avoided strong correlations between predictor variables related to a specific factor, i.e. Spearman's coefficient higher than or equal to |0.6| [39], and we selected the variable that was most significantly related to the response variable for consideration in the final model. To select the most parsimonious model we followed a forward-backward stepwise model-selection procedure using the corrected Akaike Information Criteria to compare models (AICc; [40]).

The final model was partitioned in order to enhance its explanatory capacity and improve the reliability and interpretation of multiple regressions in the presence of multicollinearity between predictors [41]. Variation partitioning procedures (see [42]) were used to estimate the variation of the final model explained independently by each factor (pure effects) and the variation explained simultaneously by two or more factors (overlaid effects) following subtraction techniques. A factor is a group of related predictors, in this study: spatio-temporal characteristics, water characteristics, meteorological indexes, and wild bird communities/vegetation structure. For details about the subtraction techniques used in this study see Alzaga et al. [43] and Acevedo et al. [44]. All statistical analyses were performed using SPSS 18.0 (SPSS Inc., Chicago, IL, USA) statistical software.

## Results

### A. Viral detection

During the 2-year period, 78 out of 4578 analysed faecal samples were positive by RRT-PCR (global prevalence of 1.7% [95% CI: 1.3% – 2.1%]). Detection rates by period and location are exposed in Tables 2 and 3. Twenty-six AIV were isolated from the 78 RRT-PCR positive samples, which means an overall recovery rate of 33.3%. In addition, HA and/or NA of three additional viruses in which the isolation was not achieved, were determined by direct PCR techniques. In total, 8 HA and 5 NA were identified. The most common subtypes were H3, H7, and N8, being the combination of H3N8 the most frequently detected. Virus subtypes, geographic origin of samples and detection period are shown in Table 4. All H5 and H7 viruses were detected from faeces of wild birds collected in wetlands from the Basque Country. The amino acid sequence of the cleavage site revealed that the H5 (PQRETR*GLF) and the H7 (PEIPKGR*GLF) strains were low pathogenic. No AIV RNA was detected in any of the collected water samples after concentration.

**Table 2:** Sampling effort and AIV detection according to the different sampling periods.

Dates	Period	Positive samples	N	Prevalence (%) ±95%CI
Sept 2007-Feb 2008	AM/W	5	592	0.8±0.7
Mar 2008-May 2008	SM	4	651	0.6±0.6
Jun-Aug 2008	BM	20	503	4.0±1.7
Sept 2008-Feb 2009	AM/W	11	1315	0.8±0.5
Mar-May 2009	SM	1	585	0.2±0.3
Jun-Aug 2009	BM	5	515	1.0±0.8
Sept-Dec 2009	AM/W	32	417	7.7±2.5
	TOTAL	78	4578	1.7±0.4

BM: Breeding/moult; AM/W: Autumn migration/wintering; SM: Spring migration.

**Table 3:** Sampling effort and AIV detection in each study site and season.

Sampling Location	BM			AM/W			SM			Total		
	Pos	N	Prev (%) ±95%CI	Pos	N	Prev (%) ±95%CI	Pos	N	Prev (%) ±95%CI	Pos	N	Prev (%) ±95%CI
1	2	67	3.0±4.0	2	553	0.3±0.5	0	292	0	4	912	0.4±0.3
2	13	46	28.2±13	30	369	8.1±2.8	1	252	0.4±0.8	44	667	6.6±0.4
3	0	140	0	0	239	0	4	125	3.2±3.0	4	504	0.8±1.9
4	1	139	0.7±1.4	2	249	0.8±1.1	0	121	0	3	509	0.6±0.8
5	1	140	0.7±1.4	5	235	2.1±1.8	0	104	0	6	479	1.2±0.6
6	1	115	0.9±1.7	3	288	1.0±1.2	0	47	0	4	450	0.9±1.0
7	7	371	1.9±1.4	6	391	1.5±1.2	0	295	0	13	1057	1.2±0.6
Total	**25**	**1018**	**2.5±0.9**	**48**	**2324**	**2.1±0.6**	**5**	**1236**	**0.4±0.3**	**78**	**4578**	**1.7±0.4**

BM: Breeding/moult; AM/W: Autumn migration/wintering; SM: Spring migration.

**Table 4:** Avian influenza virus subtypes detected during the study.

Sampling location	Period	Virus subtypes
1	BM; AM/W	H13N8 (x2), H3N8 (x1)
2	BM; AM/W	H5N2 (x1), H7N? (x8), H7N8 (x1), H3N8 (x11), H11N? (x4), H11N2 (x1)
3	n/d	n/d
4	n/d	n/d
5	AM/W	H6N1 (x2) H8N4 (x1)
6	n/d	n/d
7	BM	H4N6 (x2)

n/d: not detected; BM: Breeding/moult; AM/W: Autumn migration/wintering; SM: Spring migration.

### B. Factors determining LPAIV prevalence

**Figure 2:**
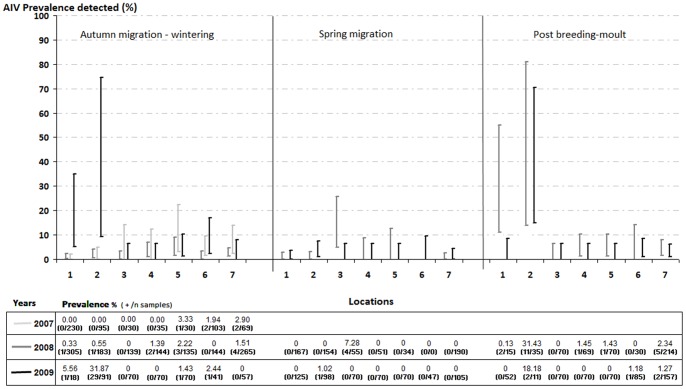
AIV prevalence in Spanish wetlands: variation of mean AIV prevalences by season, year and location (Bars reflect the 95% confidence interval).

In the exploratory stage and with regards to location and season, differences in prevalence were found among study sites (with highest detection rates in wetlands from the Basque Country) and periods (being postbreeding/moult and autumn migration/wintering those with higher probability of AIV detection) (Figure 2).

**Figure 3:**
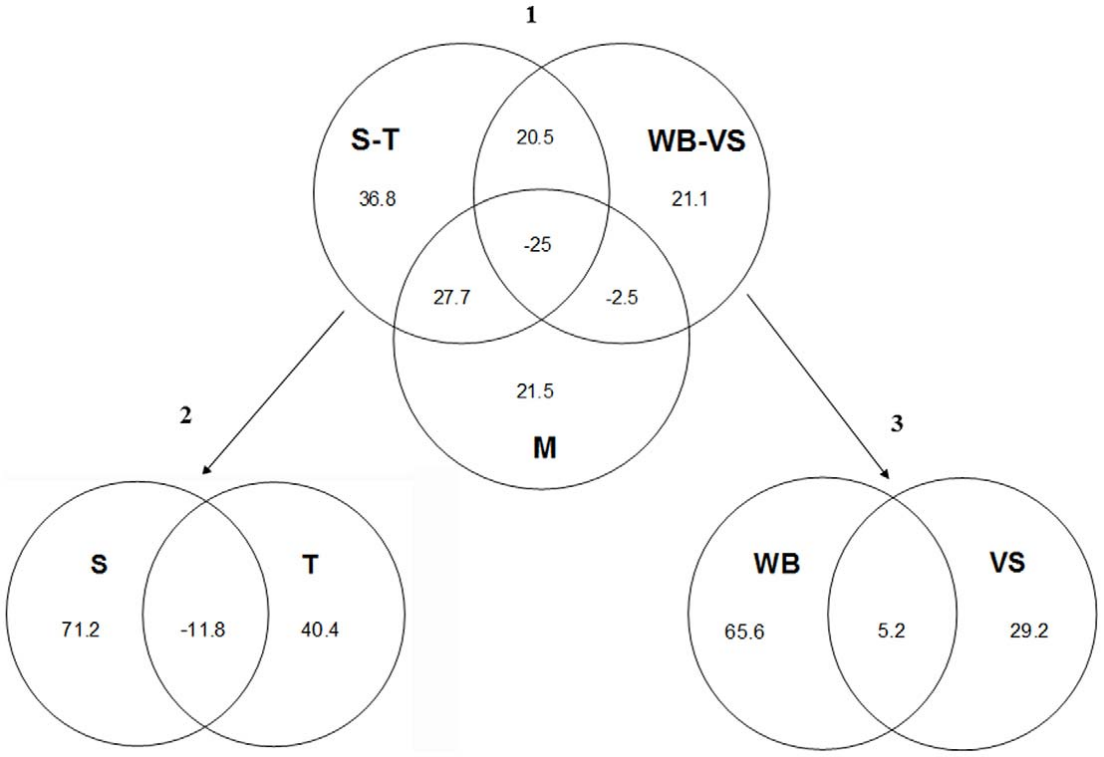
Factors implicated in AIV ecology in Spanish wetlands: results of variation partitioning of the final model in space-time (S-T), wild bird community-vegetation structure (WB-VS) and meteorological (M) factors (1), and of the partial models obtained for the space (S) – time (T) factor (2), and for the wild bird community (WB) – vegetation structure (VS) factor (3). Values shown in diagrams are the percentages of explained variation.

The final model, in which location, season and year were included as fixed variables, retained three additional predictors related with meteorological conditions, one related to vegetation structure and two related to wild bird community (see Tables 5 and 6). The model explained 16.4% of the total deviance. Variation partitioning results of the final model are shown in Figure 3. Irrespective of the other considered factors, the combination of space and time variables (location, year and period) explained the largest proportion of the variation (36.8%). In terms of pure effects, this was followed in relevance by the meteorological factor (21.5%), and finally the combination of wild bird community data and vegetation structure, which explained 21.1% of the variation (Figure 3). Partitioning of complex factors into their components demonstrated that within the variation explained by the space/time factor, the highest amount of variation was explained by “space” (71.2%), while “time” accounted for 40.4% of variation (Figure 3). As regards the combined factor wild bird community/vegetation structure (Figure 3), density and richness of wild hosts explained more variation (65.6%) than vegetation structure (29.2%).

**Table 5:** Summary of the stepwise model selection procedure based on AICc used to model avian influenza virus prevalence.

ΔAICc	AICc	Model
64.92	185.42	Null model (including location, season and year)
51.87	172.37	Total monthly rainfall (V1)
42.75	163.25	V1+ Mean monthly temperature (V2)
32.82	153.32	V1 + V2 + Monthly mean of lowest daily temperature (V3)
26.92	147.42	V1 + V2 + V3 + Vegetation thickness (V4)
9.58	130.08	V1 + V2 + V3 + V4 + Anseriformes density (V5)
0	120.50	V1 + V2 + V3 + V4 + V5 + Species richness

**Table 6:** Variables retained in the final model for avian influenza virus prevalence, their coefficients, Wald test values and significance (P-value).

Variables	Coefficient	Wald	P-value
Intersect	−2.76	5.969	0.015
Location	-	48.761	<0.001
Year	-	0.147	0.701
Period	-	19.465	<0.001
Total monthly rainfall	−0.10	9.509	0.002
Mean monthly temperature	0.70	14.237	<0.001
Monthly mean lowest daily temperature	−0.31	25.243	<0.001
Vegetation thickness	−0.23	21.359	<0.001
Anseriformes density	−0.002	19.308	<0.001
Wild bird species richness	−0.39	17.069	<0.001

## Discussion

AIV transmission and persistence among wild birds are difficult to assess because both depend on a broad variety of factors, including host community (abundance and diversity of wild birds), environmental parameters (viral tenacity in natural environments) and multiple interactions between them [45]. The multifactorial approach proposed here provides new insights on broad scale elements influencing AIV epidemiology.

Faecal sampling for AIV monitoring in wild bird populations has been suggested as a valid alternative to the more-invasive and capture dependent methods based on cloacal sampling [46]. In this study, we used faecal sampling to assess patterns of AIV prevalence. Important seasonal and geographical variations were detected, with high detection rates (up to 7.7%) at certain times and locations. However, the general prevalence 1.7% [95% CI: 1.3% – 2.1%] was lower than that detected in previous years by active surveillance in the same Spanish wetlands [17]–[19] and other Mediterranean countries [47], [48]. Our prevalence rates might also underestimate the real ones since our non invasive sampling approach did not allow the detection of AIV excreted by the respiratory tract [16].

The highest AIV prevalence was found in wetlands from the Basque Country, especially in late summer, autumn and winter (BM and AM/W). The wetlands sampled in this area are the smallest of the study as from total surface and water volume. Small waterbodies in areas with little availability of wetlands could favour AIV transmission due to elevated host densities and lower viral dilution that increase opportunity for AIV exposure [20]. In this respect, our findings might represent a relevant feature that should be considered in surveillance programs. As a fact, the concerned wetland is the one in which the only H5N1 positive case in Spain was recorded [19].

With regards to virus subtypes identified during the study, a noteworthy finding is the relatively high H7 prevalence detected (26.5%) among the identified AIV strains, as compared with previous results in wild birds in Spain [17], [19]. However, higher rates have been found in other Mediterranean countries such as Italy [49]. On the contrary, we obtained only one H5 strain (H5N2), similar to the data previously obtained in North East Spain [17] but markedly lower than H5 prevalence detected in other European countries [47], [50].

Variation partitioning procedure has been widely used in conservation biology (e.g., [51]), but it has only recently been applied to epidemiological studies [43], [44]. We consider that the application of this analytical approach to the complex AIV epidemiology in natural ecosystems can provide new and interesting insights into the relative contribution of different – related – factors underlying AIV persistence and transmission. The variation partitioning of the risk factors model identified that, independently of the other considered factors, the space/time factor was the most relevant to explain variation in AIV prevalence (36.8%). Within this factor, a higher explanatory power of “location” was obtained, which is substantially higher than that attained by “season”. This result may agree with the virological findings obtained in one of the sampled locations (Salburua), where much higher prevalences were consistently found as compared with the rest of wetlands, especially during BM and AM/W.

Regarding the effect of the temporal factor, numerous studies have previously evidenced seasonal and interannual fluctuations in AIV dynamics [24], [50]. In this study, the highest prevalence rates were obtained during AM/W in 2009 (7.7%) in consistence with what is generally reported for Europe. However, important between-years fluctuations were detected for this season (AIV prevalence as low as 0.85% in 2007 and 0.83% in 2008), which highlights the relevance of long-term studies that allow gaining a comprehensive unbiased knowledge of AIV dynamics in natural ecosystems [52]. Considering data from all the years included in the study, overall prevalence was higher during BM season 2.5% [95% CI: 1.5% – 3.3%], followed by AM/W 2.1% [95% CI: 1.4% – 2.6%]. Higher detection rates obtained during these periods have been related to the influx of young immunologically naïve birds that are highly susceptible to infection [50]. By contrast, prevalence during SM was significantly lower 0.4% [95% CI: 0.1% – 0.7%]. Although AIV incidence during AM/W was lower than in previous research carried out in some of the sampled locations [17], [18] and other countries in Europe [49], it was significantly higher than the prevalence observed during spring. Autumn migrants that arrive in Spain during Sept-Oct could introduce new AIV strains, favouring transmission among wild birds that concentrate in high numbers in their wintering grounds in Southern Europe. The important role of Mediterranean wintering areas in AIV epidemiology has previously been suggested [18], [47], [53].

The pure effect of the meteorological factor was the next in importance in explaining AIV prevalence, being retained in the final model both precipitation and temperature indexes. These results are in accordance with previous studies, since temperature and humidity levels have been recognized as critical parameters on the environmental tenacity of AIV [8], [18]. The influence of climatic conditions on AIV prevalence implies a predominant role of environmental transmission (via persisting virus in water, faeces and other surfaces), as previously suggested in other boreal and temperate regions [5], [12], [20]. By contrast, in tropical ecosystems, AIV prevalence has been associated to wildfowl density with no influence of climatic conditions, which implies a prevailing role of direct transmission via the respiratory route or through recently shed virus in the environment [16].

Finally, the factor related with wild bird communities and vegetation structure had a smaller contribution to the explanation of total variation. Within this factor, main explanatory power was attributed to wild hosts (i.e. density and richness of wild birds). The presence of suitable hosts (wild birds), and especially those from the orders Anseriformes that are considered the main reservoir of AIV, is essential for viral transmission and environmental perpetuation in the ecosystem [45]. A positive association between AIV prevalence and wildfowl density has also been observed in African wetlands [16].

The vegetation structure barely explained the model's deviance, showing a limited pure effect. Vegetation thickness, that was the only variable of this factor retained in the final model, was included in the analysis as a measure of shelter availability for wild birds. Presence of dense vegetation around wetlands, offering shelter and food for birds, would potentially lead to higher density and aggregation of hosts [34], favouring AIV transmission [35].

The essential role of water-borne transmission in AIV epidemiology has been widely proven by experimental and field studies [8], [54], [55] and more recently through mathematical modelling works [11], [12], [20]. AIV has been detected in surface waters in areas with high duck densities, and generally low temperatures, even without water concentration [56]. Recent studies have also shown that AIV water persistence data from laboratory trials are largely modified by real world conditions in water bodies, especially as conditions are not stable over time [5]. In this study it was not possible to detect AIV genome in surface water samples. This could be due to the effect of field conditions affecting AIV persistence in Spanish water bodies, such as UV exposure that is still largely unexplored [5]. On the other hand, the water volume collected in every visit (4 litres per wetland) might be not large enough for detection of low level of AIV contamination. Also virus degradation caused by water microbiota in natural environments should not be neglected. When water sampling and processing was performed, there was no single recommended standardized method for recovering or detecting these viruses from water samples [5], [57]. However, a concentration method for AIV detection from large volumes of surface water has been very recently developed and validated by Deboosere et al. [58]. Unfortunately, we had no more water samples available to repeat analyses using this new methodology.

None of the variables included in the “water” factor were retained in the final model, even though the abiotic parameters registered in every visit (pH, temperature, salinity and turbidity of water) are thought to be the main determinants of virus survival in aquatic ecosystems [5], [8], [57], [59]. In contrast to controlled laboratory conditions, in our study, virus influx, dilution and other biotic factors could potentially have been more important than the degree of variation of the tested physical properties [5].

In conclusion, the results of this work are useful to better understand the ecological drivers that may modulate the occurrence of AIV in wetlands. The integrated approach presented in this study can be applied to different epidemiological scenarios and provide useful guidelines for AIV risk assessment, identifying potential hotspots of AIV activity and contributing to optimize surveillance systems in wild birds.

## Supporting Information

Table S1 **Average and range values of water parameters by sampling period and location.**
(DOCX)
