# Supplementary material for: Ecological Factors Driving Avian Influenza Virus Dynamics in Spanish Wetland Ecosystems
Source: PLoS One. 2012 Nov 12;7(11):e46418. doi: 10.1371/journal.pone.0046418 (PMC3495955; doi:10.1371/journal.pone.0046418)
Supplement: Table S1 — Average and range values of water parameters by sampling period and location. (DOCX) [file pone.0046418.s001.docx]

| **Sampling location** | **Period** | **Temperature**  **(ºC)** | | **pH** | | **Conductivity**  **(Ms/cm)** | | **Turbidity**  **(FTU)** | |
| --- | --- | --- | --- | --- | --- | --- | --- | --- | --- |
|  |  | **Average** | **Range** | **Average** | **Range** | **Average** | **Range** | **Average** | **Range** |
| **1** | **AM/W** | 16.7 | 10.2-23.5 | 8.0 | 7.6-8.5 | 44.7 | 37.8-50.2 | 5.31 | 3.3-8.3 |
|  | **SM** | 14.8 | 14.7-14.9 | 7.7 | 7.7-7.8 | 16.2 | 1.7-30.8 | 68.4 | 10.8-126 |
|  | **BM** | 26.1 | 24.9-27.2 | 7.8 | 7.3-8.2 | 32.6 | 30.3-34.9 | 24.0 | 18.8-29.3 |
| **2** | **AM/W** | 10.8 | 6.9-16.3 | 7.6 | 7.4-7.9 | 0.5 | 0.5-0.5 | 23.3 | 5.4-32.8 |
|  | **SM** | 9.1 | 7.2-11.0 | 7.5 | 7.5-7.5 | 0.5 | 0.4-0.5 | 10.6 | 5.0-16.2 |
|  | **BM** | 21.8 | 18.6-25.0 | 7.5 | 7.4-7.6 | 0.5 | 0.5-0.5 | 65.3 | 23.3-107.4 |
| **3** | **AM/W** | 17.2 | 12.0-19.8 | 7.7 | 7.3-8.2 | 5.2 | 1.4-8.7 | 46.1 | 27.1-75.9 |
|  | **SM** | 21.7 | 21.4-22.0 | 8.3 | 7.8-8.8 | 3.8 | 0.9-6.7 | 59.7 | 37.0-82.0 |
|  | **BM** | 26.5 | 24.8-28.3 | 7.9 | 7.8-8.0 | 1.3 | 1.2-1.4 | 69.8 | 54.4-85.1 |
| **4** | **AM/W** | 18.1 | 11.8-21.6 | 7.8 | 7.2-8.4 | 16.7 | 5.6-48.8 | 53.8 | 15.8-111.7 |
|  | **SM** | 19.1 | 17.6-20.7 | 7.8 | 7.4-8.3 | 18.3 | 16.6-20.0 | 90.7 | 80.0-101.2 |
|  | **BM** | 28.0 | 21.6-34.4 | 8.4 | 7.4-9.3 | 16.4 | 16.4-16.5 | 38.2 | 28.0-48.3 |
| **5** | **AM/W** | 21.0 | 14.5-27.4 | 7.7 | 6.9-8.2 | 324.0 | 123-855.3 | 65.3 | 16.8-125.2 |
|  | **SM** | 26.2 | 20.4-32.0 | 8.2 | 7.9-8.5 | 139.5 | 129.6-149.3 | 24.0 | 11.0-37.1 |
|  | **BM** | 28.8 | 26.0-31.6 | 8.0 | 7.9-8.2 | 125.0 | 119.2-130.7 | 72.9 | 35.9-109.9 |
| **6** | **AM/W** | 15.4 | 6.5-23.8 | 8.3 | 7.9-8.9 | 1.6 | 1.4-1.7 | 84.2 | 33.0-170.7 |
|  | **SM** | 18.3 | 16.0-20.6 | 9.0 | 8.4-9.5 | 1.9 | 1.7-2.0 | 155.7 | 66.7-244.7 |
|  | **BM** | 28.0 | 25.8-30.2 | 8.5 | 8.3-8.6 | 2.0 | 1.7-2.3 | 153.2 | 83.2-223.5 |
| **7** | **AM/W** | 15.9 | 7.8-26.1 | 8.9 | 8.7-9.1 | 3.1 | 2.9-3.4 | 42.6 | 21.1-74.0 |
|  | **SM** | 19.4 | 18.0-20.8 | 8.6 | 8.4-8.7 | 2.9 | 2.2-3.5 | 99.9 | 85.1-114.7 |
|  | **BM** | 30.8 | 29.7-31.9 | 8.9 | 8.9-8.9 | 17.5 | 16.1-19.0 | 178.8 | 143.0-214.0 |

Table S1
